# Untargeted metabolomics reveals transformation pathways and metabolic response of the earthworm *Perionyx excavatus* after exposure to triphenyl phosphate

**DOI:** 10.1038/s41598-018-34814-9

**Published:** 2018-11-06

**Authors:** Lei Wang, Xulei Huang, Anna Karen Carrasco Laserna, Sam Fong Yau Li

**Affiliations:** 1 Department of Chemistry, National University of Singapore, Singapore, 3 Science Drive 3, Singapore; 2 NUS Environmental Research Institute (NERI), #02-01, T-Lab Building (TL), 5A Engineering Drive 1, Singapore, 117411 Singapore

## Abstract

Triphenyl phosphate (TPHP) is one of the most highly utilized organophosphorus flame retardants, and has been frequently detected in various environmental matrices, including soil. So far, limited information is known regarding the potential toxicity of TPHP to the earthworm-soil ecosystem. We investigated the metabolism of TPHP and the perturbation of the endogenous metabolome in the earthworm, *Perionyx excavatus*, using gas chromatography mass spectrometry (GC-MS) and liquid chromatography quadrupole time-of-flight (LC-QTOF)-based untargeted metabolomics approach after acute exposure to TPHP for one and two days through a filter paper contact test, as well as after chronic exposure for 28 days in a soil microcosm experiment. TPHP showed low bioaccumulation potential in the earthworm-soil ecosystem at concentrations of 10 mg/kg and 50 mg/kg. Identified phase I metabolites include diphenyl phosphate, mono-hydroxylated and di-hydroxylated TPHP. Two groups of phase II metabolites, thiol conjugates (including mercaptolactic acid, cysteine, cysteinylglycine, and mercaptoethanol conjugates) and glucoside conjugates (including glucoside, glucoside-phosphate, and C_14_H_19_O_10_P conjugates), were putatively identified. Only acute TPHP exposure caused significant perturbations of the endogenous metabolome in earthworms, featuring fluctuations in amino acids, glucose, inosine and phospholipids. These results reveal novel phase II metabolism and toxicity of TPHP in *P. excavatus*.

## Introduction

Flame retardants are commonly added to manufactured materials, such as textiles and plastics, to inhibit the spread of fire. Since the phase-out of persistent, bioaccumulative and toxic polybrominated diphenyl ethers in 2004, the production of alternative flame retardants, such as organophosphate esters, has been increased to meet fire safety standards^1^. Triphenyl phosphate (TPHP) is one of the most highly utilized organophosphorus flame retardants, with usage volume reaching 4500–22700 tons in the United States as of 2006^2^. It is incorporated in polyvinyl chloride and unsaturated polyester resins, as well as in commercial mixtures, such as Firemaster 550 (FM550)^3^. Since many flame retardants are present as additives rather than chemically bonded to the consumer products, they can be readily released into the environment via volatilization, leaching and abrasion^1^. TPHP has been detected in various environmental matrices. The concentrations of TPHP in private house dust samples reached 7360 ng/g (geometric mean) in 2002–2007^4^, and 3000 ng/g (median value) in 2006–2011^5^ in USA. A high level of TPHP (12,000 pg/m^3^) originating from traffic emissions was reported in the remote air from northern Finland^6^. The maximum reported level of TPHP reached 14 μg/L in the influent of wastewater treatment plant in Norway in 2007^7^. Since TPHP is widely distributed in the environment, there is growing interest in its metabolic fate and toxicological effect on ecological systems.

Metabolism of TPHP has previously been studied *in vitro* in chicken embryonic hepatocytes^8,9^, human liver microsomes and the S9 fraction^10^ and human serum^11^, as well as *in vivo* in zebrafish^12^. Major phase I metabolites are diphenyl phosphate (DPHP), mono-hydroxylated (para- and meta-OH-TPHP) TPHP, and di-hydroxylated TPHP (di-OH-TPHP). Reported phase II metabolites include the glucuronide and sulfate conjugates of OH-TPHP as well as the glucuronide conjugate of di-OH-TPHP. Due to the structural similarity of TPHP with organophosphorus pesticides, TPHP has been linked with neurotoxicity, but so far reported studies have been conflicting^1^. Recent studies revealed that TPHP possesses endocrine disrupting properties^13–17^, and can also induce developmental toxicity in fish, including neurotoxicity^18^ and cardiotoxicity^3^. In rats, it can cause serum hypertriglyceridemia by inhibiting specific liver carboxylesterases^19^.

Knowledge of TPHP metabolism has so far been gained only from studies of human, chicken and aquatic organism exposure, whereas toxicological information available refers only to *in vitro* assays or *in vivo* studies involving rats and aquatic organisms. So far, no work regarding the metabolism and toxicity of TPHP on terrestrial organisms, such as earthworms, has been reported. Soil is vulnerable to contamination originated from aqueous TPHP, as well as atmospheric TPHP that can be globally transported and then deposited/precipitated^1^. Moreover, considerable release of TPHP to soil from e-waste recycling activities^20^ and plastic waste treatment^21^ has been confirmed. Once TPHP enters into the soil, its reverse release from soil to the air is considered to be negligible, due to its high estimated soil sorption coefficient (log K_oc_ = 3.72) and low vapor pressure (1.2 × 10^−6^ mm Hg at 25 °C)^1^. These facts raise the concerns on whether TPHP elicits adverse effects on soil-dwelling organisms. Earthworms play important roles in organic matter turnover in terrestrial ecosystems, and are frequently used in ecotoxicology to investigate toxicity of anthropogenic chemicals present in soil^22^. Thus, investigating the metabolic fate and toxicity of TPHP on earthworms is warranted.

Metabolomics is an “omics” technology that characterizes measurement of the perturbations of a variety of metabolites in cells, tissues and biofluids under aberrant conditions. By analyzing significantly differentiated metabolites and their related pathways, metabolomics provides the opportunity to potentially gain insights on novel toxicity targets for xenobiotics. This saves time and resources compared with using multiple targeted assays in traditional toxicology studies, and allows discovery of novel biomarkers^23^. A few typical works^24–26^ using metabolomics to study sub-lethal toxicity of environmental contaminants on earthworm have exemplified its utility. A recent work using metabolomics to study zebrafish exposure to TPHP provided insights on the perturbation of carbohydrate and lipid metabolism^27^.

In this study, earthworms were exposed to TPHP in both acute and chronic exposure scenarios to investigate TPHP metabolism and toxicity. TPHP metabolites and endogenous metabolites of earthworm were comprehensively profiled by using multi-platform techniques combing liquid chromatography - quadrupole time-of-flight (LC-QTOF) tandem mass spectrometry and gas chromatography - mass spectrometry (GC-MS) analyses. Significantly altered metabolites were identified through multivariate statistical analysis. Various biotransformation products of TPHP were identified and perturbations of certain metabolic pathways caused by TPHP exposure were proposed.

## Results

### TPHP degradation in soil and accumulation in earthworm

**Figure 1:**
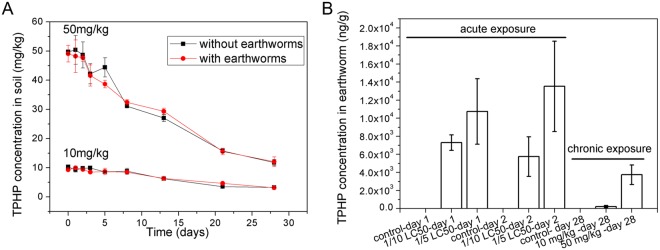
Concentrations of TPHP (**A**) in soil with and without earthworms through 28 days and (**B**) in earthworms after acute and chronic exposure on a dry weight basis. Note that acute and chronic exposures refer to filter paper and soil experiments, respectively. In acute exposure, the 1/10 LC_50_ and 1/5 LC_50_ concentrations equal to 0.425 and 0.85 μg/cm^2^, respectively. Data is shown with mean and associated standard derivation.

Figure 1(A) shows TPHP concentrations in soil through 28 days of chronic exposure. For both exposure groups of 50 mg/kg and 10 mg/kg, TPHP concentrations decreased gradually through time, both with earthworms and without earthworms in the soil. Final concentrations reached around 1/4 of the initial concentration of 50 mg/kg and 1/2 of the initial concentration of 10 mg/kg. Su *et al*.^28^ revealed that TPHP maintained its concentration in aqueous solution at pH 7–11 in the dark for 35 days, thus it is likely that TPHP did not degrade in the soil pore water. As the soil was placed in dark conditions during experiments, the concentration decrease may be mostly attributed to activity of soil microorganisms. An earlier study also revealed that TPHP degradation in soil was mostly mediated by microorganisms^29^.

TPHP concentrations in earthworms after acute and chronic exposure are shown in Fig. 1(B). For acute exposure, average TPHP concentrations in earthworms exposed to 1/10 LC_50_ for one day was higher than for two days, indicating fast biotransformation rate. The body-burden of TPHP depends on exposure concentrations. The bioaccumulation factor (BAF) can be calculated by dividing TPHP concentration in worms C_worm_ (ng/g_dry weight_) with that in the soil C_soil_ (ng/g_dry weight_). Here, BAF was calculated as 0.022–0.069 for 10 mg/kg group, and 0.075–0.319 for 50 mg/kg group. The BAF values are all less than 1, indicating low risk of bioaccumulation in earthworm-related food webs.

### Identification of TPHP metabolites

**Figure 2:**
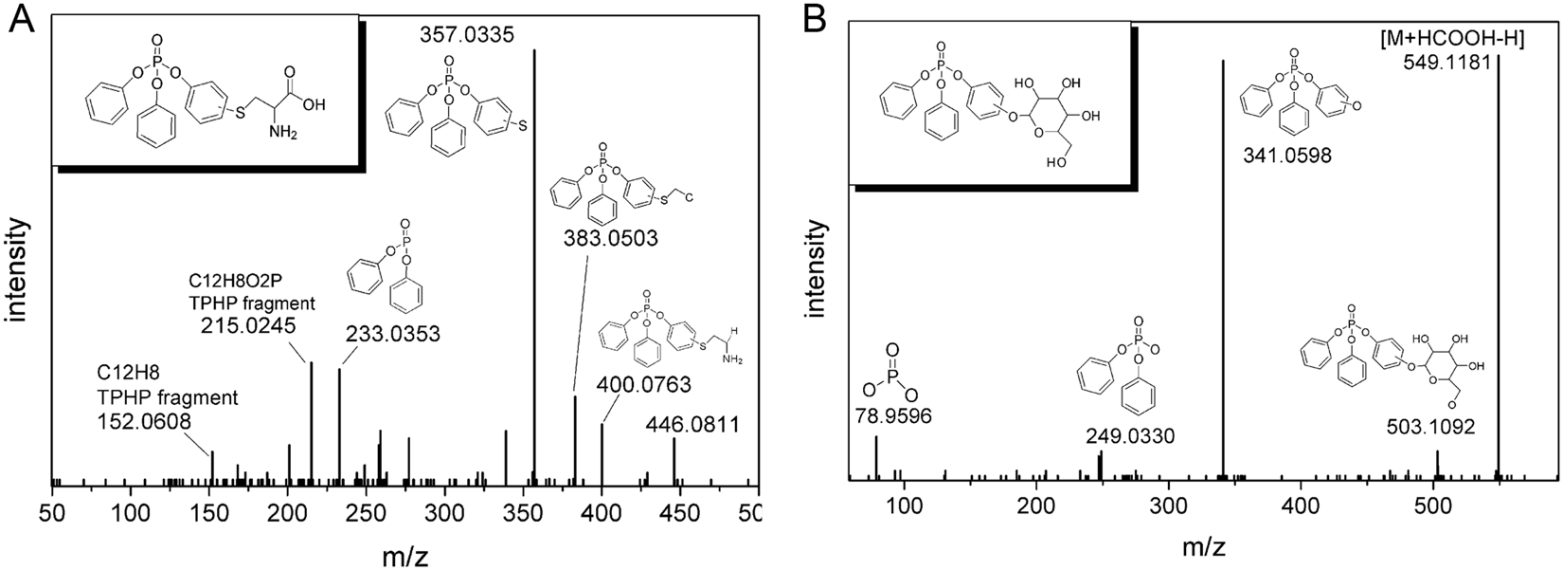
MS/MS spectrum of (**A**) cysteine-TPHP (*m/z* 446.0811 in ESI+) and (**B**) glucoside-TPHP (*m/z* 549.1181 in ESI-) with annotation of the fragments. M refers to the intact metabolite.

In soil without earthworms, we identified two TPHP transformation products, DPHP and OH-TPHP. We putatively identified a list of phase I and phase II metabolites, as shown in Table 1, from the earthworm body after 28 days of exposure. Phase I metabolites include DPHP, OH-TPHP and di-OH-TPHP, which are consistent with previous reports of TPHP metabolites^8–12^. We did not find OH-DPHP, or monophenyl phosphate, indicating that no further hydroxylation or hydrolysis occurred. Interestingly, we found two groups of possible phase II conjugates, i.e. thiol conjugates and glucoside conjugates. Most of them are OH-TPHP conjugates, and there was only one di-OH-TPHP conjugate. The thiol conjugates included mercaptolactic acid, cysteine, cysteinylglycine, and mercaptoethanol conjugates, while glucoside conjugates included glucoside, glucoside-phosphate and C_14_H_19_O_10_P conjugates. The fragment with *m/z* 383.0503 appeared in mercaptolactic acid, cysteine, and cysteinylglycine conjugates, and we assigned it as TPHP-S_1_CH_2_C_1_. This assignment can be supported by the ESI+ Q-TOF fragments of L-cysteine, which can be found in the METLIN database. L-cysteine has a fragment with *m/z* 58.9956, which is assigned as H_1_S_1_CH_2_C_1_ in METLIN. This structure is the same as the conjugated part of TPHP-S_1_CH_2_C_1_, given that one hydrogen has been used for dehydration condensation in the conjugation process. We assigned the fragments with *m/z* 152.0608 and 215.0245 based on calculated formulae. Using a criterion of mass error <15 ppm and elemental compositions range of C_0–100_ H_0–100_ O_0–10_ P_0–1_, the predicted formulae for 152.0608 were C_5_H_13_O_3_P and C_12_H_8_, and the predicted formula for 215.0245 is C_12_H_8_O_2_P. C_5_H_13_O_3_P has an unsaturation of zero, which is not reasonable as a TPHP fragment. Therefore, C_12_H_8_ and C_12_H_8_O_2_P were annotated for *m/z* 152.0608 and 215.0245, respectively. In fact, the two fragments are from both triphenyl phosphate and diphenyl phosphate, and hence their assignment does not affect the identification of TPHP conjugates. The fragment with *m/z* 241.0118 in the MS/MS spectra of *m/z* 583.0797 in ESI- was assigned as C_14_H_19_O_10_P, i.e. a structure with missing hydrogen in the carbon that is bonded to TPHP-O. This structure might be explained by the loss of TPHP-OH from the precursor ion in the collision process. The structure of C_14_H_19_O_10_P could not be unambiguously identified by MS/MS spectra, and was tentatively assigned in Supplementary Fig. S2. The ion of the glutathione conjugate (a typical thiol conjugate) of OH-TPHP was found at much lower abundance than other thiol conjugates. Its MS/MS was not even triggered by information dependent acquisition. This indicates that glutathione conjugation comprises only a small portion of the thiol conjugations in the phase II metabolism of TPHP by earthworms. OH-TPHP and its thiol conjugates all have two or three isomers eluting at different retention times. This indicates that the hydroxyl substitution of TPHP could occur at the ortho-, meta-, and para- positions. Our results add to previously reported TPHP hydroxylations by Nele Van den *et al*.^10^, who found para- and meta- hydroxylated metabolites of TPHP in human liver fractions. Different glucoside isomers could also derive from alpha and beta glycosidic conjugation, which was reported previously in earthworms^30^. The MS/MS spectra of cysteine-TPHP (*m/z* 446.0811 in ESI+) and glucoside-TPHP (*m/z* 549.1181 in ESI-) are shown in Fig. 2 for structure confirmation. The MS/MS spectra of other conjugates can be found in Supplementary Fig. S2.

**Table 1:** Identified TPHP metabolites in earthworms from LC-QTOF spectra after 28 days of TPHP exposure in soil.

Type	Metabolites	Adducts	Formula	Theoretical *m/z*	Experimental *m/z*	Mass error (ppm)	Theoretical isotope ratio (%)	Experimental isotope ratio (%)	Retention time (min)	Characteristic product ions
phase I metabolites	DPHP	[M + H]^+^	C_12_H_12_O_4_P_1_	251.0468	251.0458	3.7	13.6	13.5	10.52	152.0608, 175.0128, 215.0245, 233.0353
	OH-TPHP (3 isomers)	[M + H]^+^	C_18_H_16_O_5_P_1_	343.0730	343.0726	1.2	20.4	19.5	12.27, 13.95, 16.72	152.0608, 215.0245, 233.0353, 357.0335
		[M-H]^−^	C_18_H_14_O_5_P_1_	341.0584	341.0596	3.3	20.4	20.6	13.49, 16.71, 17.10	93.0345, 185.0614, 233.0383, 249.0347
	di-OH-TPHP (4 isomers)	[M + H]^+^	C_18_H_16_O_6_P_1_	359.0679	359.0680	0.2	20.4	19.5	12.85, 13.07, 13.43, 16.22	153.0709, 215.0245, 233.0353, 251.0456
thiol conjugates	mercaptolactic acid-TPHP (3 isomers)	[M + H]^+^	C_21_H_20_O_7_S_1_P_1_	447.0662	447.0656	1.3	24.6	22.6	13.14, 13.94, 16.24	152.0608, 215.0245, 233.0353, 357.0335
		[M + NH_4_]^+^	C_21_H_23_O_7_N_1_S_1_P_1_	464.0927	464.0925	0.5	25.0	30.0	12.08, 13.91, 16.25	152.0608, 215.0245, 233.0353, 357.0335
		[M-H]^−^	C_21_H_18_O_7_S_1_P_1_	445.0516	445.0518	0.4	24.6	24.6	13.38,15.64	177.0355, 249.0347, 267.1975, 357.0363
	cysteine-TPHP (2 isomers)	[M + H]^+^	C_21_H_21_O_6_N_1_S_1_P_1_	446.0822	446.0813	2.0	25.0	23.8	12.05, 13.15	152.0608, 215.0245, 233.0353, 357.0335
	cysteinylglycine-TPHP (2 isomers)	[M + H]^+^	C_23_H_24_O_7_N_2_S_1_P_1_	503.1036	503.1028	1.7	27.6	30.5	11.85, 12.80	152.0608, 215.0245, 233.0353, 357.0335
	mercaptoethanol-TPHP (2 isomers)	[M + H]^+^	C_20_H_20_O_5_S_1_P_1_	403.0764	403.0762	0.1	23.4	25.1	14.69, 17.45	152.0608, 215.0245, 233.0353, 357.0335
	glutathione-TPHP	[M+H]^+^	C_28_H_31_O_10_N_3_P_1_S_1_	632.1462	632.1444	2.9	33.7	30.5	13.06	—
glucoside conjugates	glucoside-TPHP	[M+NH_4_]^+^	C_24_H_29_O_10_N_1_P_1_	522.1524	522.1511	2.4	27.8	24.4	13.55	343.0732
		[M+COOH]^−^	C_25_H_26_O_12_P_1_	549.1167	549.1186	3.4	28.5	27.6	13.57	233.0366, 249.0330, 341.0598, 503.1092
	phosphate-glucoside-TPHP	[M+H]^+^	C_24_H_27_O_13_P_2_	585.0921	585.0911	1.8	27.5	23.7	13.11	343.0732
		[M-H]^−^	C_24_H_25_O_13_P_2_	583.0776	583.0786	1.7	27.5	27.5	12.66	78.9586, 241.0018, 291.0917, 537.3042
	C_14_H_19_O_10_P-TPHP (3 isomers)	[M+H]^+^	C_32_H_33_O_14_P_2_	703.1340	703.1327	1.9	36.5	34.8	15.67, 16.31	91.0534, 127.0385, 263.0913, 343.0732
		[M-H]^−^	C_32_H_31_O_14_ P_2_	701.1195	701.1221	3.8	36.5	35.0	14.66, 15.16	78.9586, 223.0013, 359.0560, 583.0755
		[M+NH_4_]^+^	C_32_H_36_O_14_N_1_P_2_	720.1606	720.1578	3.9	36.9	39.2	14.72, 14.95, 16.30	127.0385, 263.0913, 343.0732, 703.1316
	C_14_H_19_O_10_P-TPHP-OH (3 isomers)	[M+H]^+^	C_32_H_33_O_15_P_2_	719.1289	719.1271	2.6	36.5	38.1	14.13, 14.72, 16.24	107.0478, 279.0848, 343.0732, 469.1023
		[M-H]^−^	C_32_H_31_O_15_P_2_	717.1144	717.1175	4.3	36.5	37.0	13.46, 13.98, 15.13	78.9586, 359.0560

The structure of C_14_H_19_O_10_P could not be unambiguously identified by MS/MS spectra, and are tentatively proposed in Supplementary Fig. S2.

### Multivariate analyses of GC-MS and LC-QTOF data

After acute exposure experiments, one worm died at 1/5 LC_50_ concentration after both days. The dead worms exhibited tapering tail and bleeding tissues. In contrast, there were no dead or deformed worm after chronic exposure. GC-MS and LC-QTOF in positive and negative mode were used to profile global metabolites in earthworm after acute and chronic TPHP exposure. Principal component analysis (PCA) was first performed for an overview of possible clustering of different treatment groups. Scores plot of features extracted from LC-QTOF positive mode spectra after acute exposure (Supplementary Fig. S3A) reveals a slight shift from control groups to exposure groups at both days. In addition, there were large overlaps of score points for both control groups and exposure groups at the same day. Hence, in further partial least squares discriminant analysis (PLS-DA) of these features, we grouped samples from two days together as “Control” and “Exposure”, respectively. In scores plots of features extracted from LC-QTOF negative mode spectra (Supplementary Fig. S3B) and GC-MS spectra (Supplementary Fig. S3C), we did not observe clear trends in separations between control and exposure groups. However, the samples from the 1/5 LC_50_-day 2 group were found to be clustering slightly separated from the other groups in the GC-MS dataset scores plot. This group was further compared with control-day2 group in PLS-DA. The PCA scores plots after chronic exposure using features from the three datasets (Supplementary Fig. S4) reveals large overlap for all three groups.

**Figure 3:**
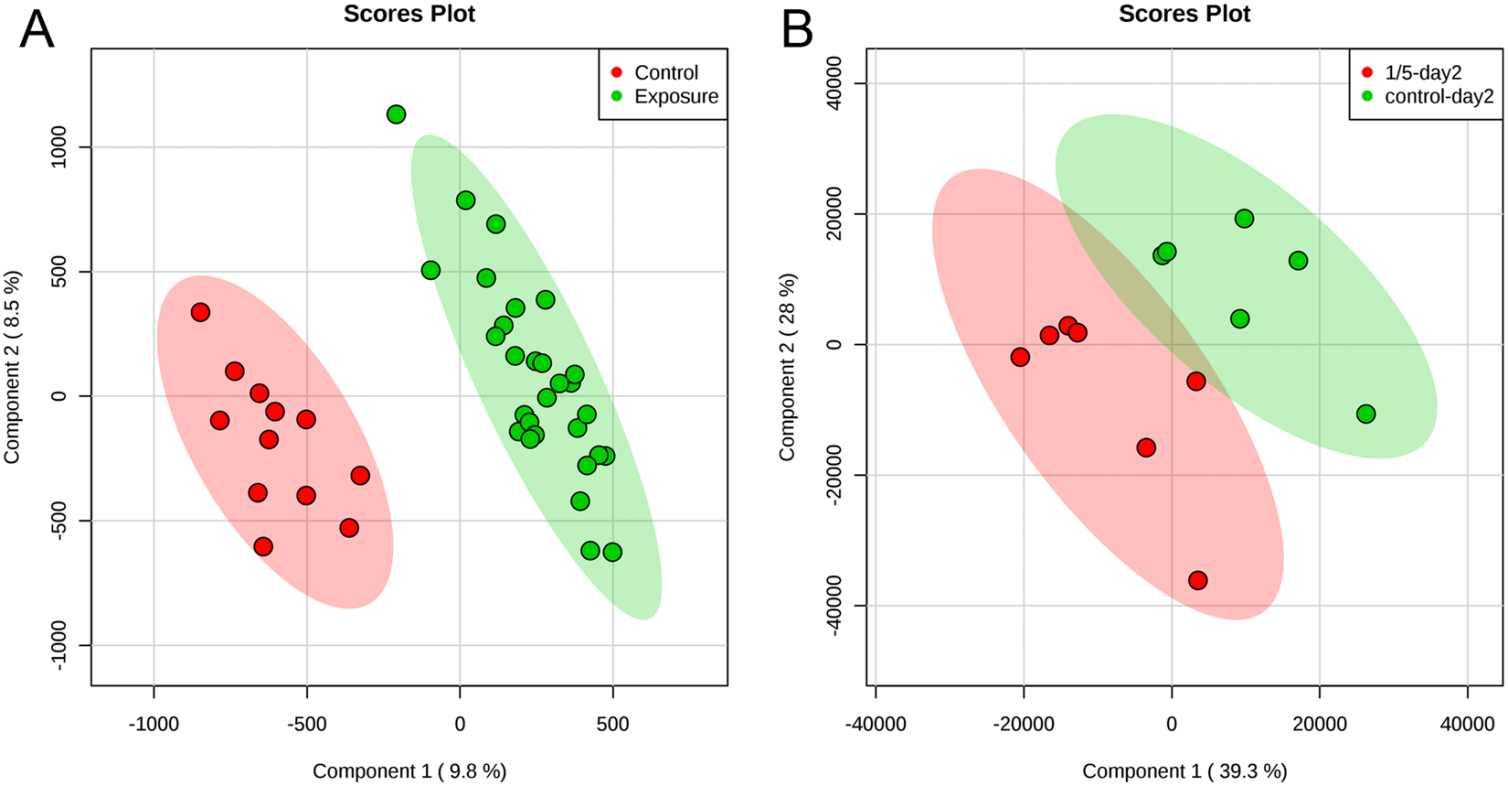
Score plot of partial least squares discriminant analysis using features from (**A**) LC-QTOF spectra (Control versus Exposure) and (**B**) GC-MS spectra (control-day 2 versus 1/5 LC_50_-day2) after acute exposure.

Observations from PCA were further examined by cross validation of PLS-DA model. A good model should have high values of model prediction accuracy as well as high values of R^2^ and Q^2^, representing the explained variance and the quality assessment of the model, respectively^31^. In consistency with PCA observations, the PLS-DA models using “Control versus Exposure” in LC-QTOF positive mode and “control day2 versus 1/5LC_50_ day2” in GC-MS generate high accuracy, R^2^ and Q^2^ values, whereas models using datasets after chronic exposure result in lower values (Table 2). Scores plots exhibit good separations between these groups after acute exposure (Fig. 3). Permutation tests were further conducted for the PLS-DA models. The significance level was 0.02 for LC-QTOF spectra (control versus exposure) and 0.01 for GC-MS spectra (control-day 2 versus 1/5 LC_50_-day2) at permutation numbers of 100. Both values were smaller than 0.05, thus meeting the criteria of significance level (*P* < 0.05). In addition, for GC-MS data between control-day 2 and 1/5 LC_50_-day 2, the scatter plot showed even clearer separation between control group and exposure group after probabilistic quotient normalization than that obtained without probabilistic quotient normalization (Fig. S5). This clearly indicates that there are differences between the two groups. This result is consistent with earthworm mortality data after exposure, suggesting considerable and negligible perturbation to endogenous metabolites after acute and chronic exposure of TPHP, respectively.

**Table 2:** Cross validation results of partial least squares discriminant analysis model from LC-QTOF and GC-MS spectra after acute and chronic exposure using two components.

Exposure	Spectra	Accurary	R^2^	Q^2^	Groups
acute exposure	LC-QTOF positive mode	0.96	0.95	0.79	Control versus Exposure
	GC-MS	0.85	0.83	0.40	Control day2 versus 1/5LC_50_ day2
chronic exposure	LC-QTOF positive mode	0.26	0.77	−0.35	Control versus 10 mg/kg versus 50 mg/kg
	LC-QTOF positive mode	0.65	0.78	−0.02	Control versus Exposure
	LC-QTOF negative mode	0.30	0.81	−0.20	Control versus 10 mg/kg versus 50 mg/kg
	LC-QTOF negative mode	0.61	0.84	0.01	Control versus Exposure
	GC-MS	0.52	0.85	0.44	Control versus 10 mg/kg versus 50 mg/kg
	GC-MS	0.74	0.74	0.17	Control versus Exposure

### Changes of endogenous metabolites after acute exposure

**Figure 4:**
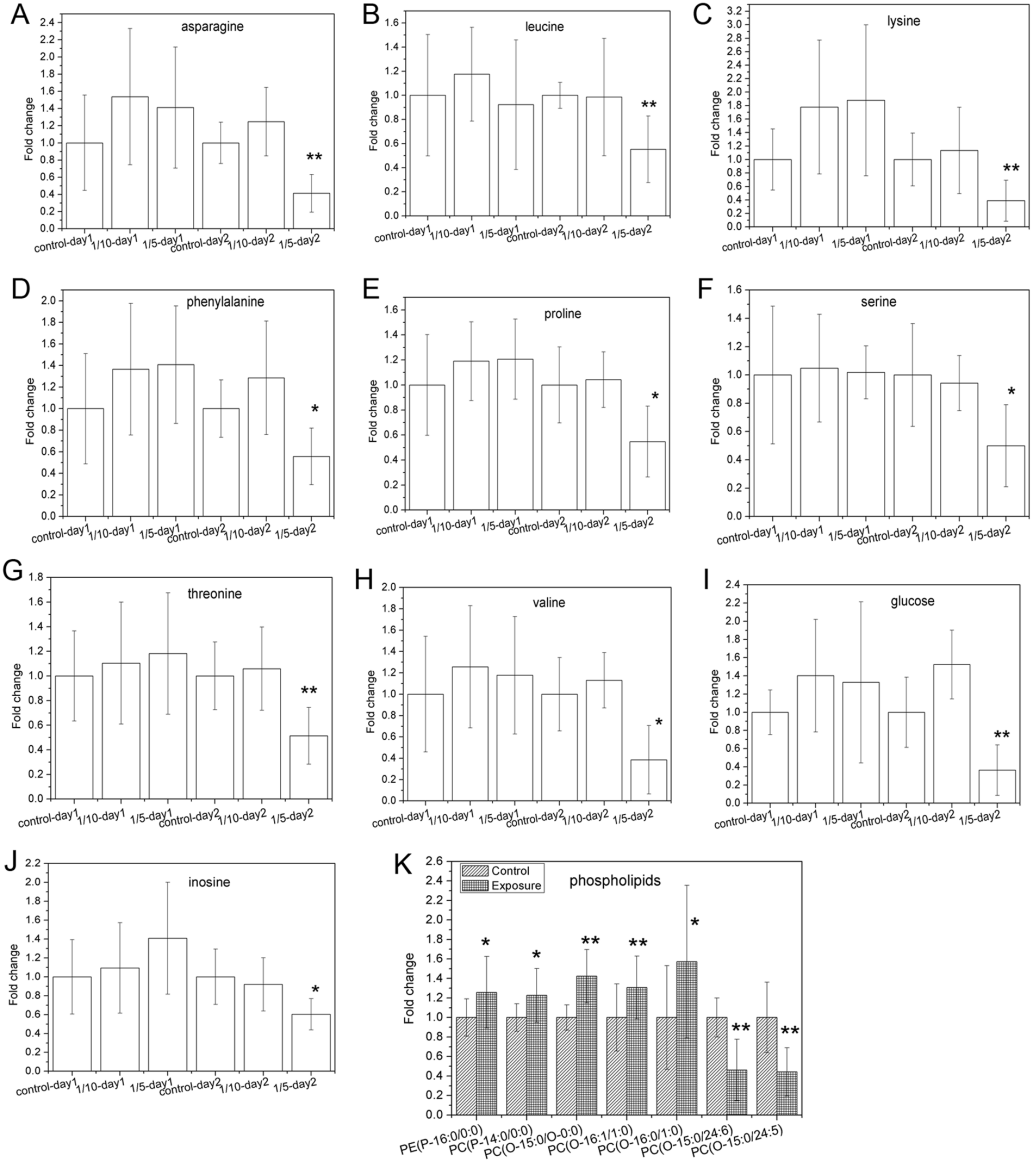
Fold changes of differentiated metabolites after acute TPHP expousre: (**A**) asparagine, (**B**) leucine, (**C**) lysine, (**D**) phenylalanine, (**E**) proline, (**F**) serine, (**G**) threonine, (**H**) valine, (**I**) glucoside, (**J**) inosine and (**K**) phospholipids. For A-J, fold changes were calculated by dividing the peak areas of the exposure groups with the mean peak area of the control group for the same day from the GC-MS chromatograms. For phospholipids, control group, “Control” refers to control-day 1 and day 2 samples, whereas “Exposure” refers to the pool of the rest of the samples, and fold changes were calculated by dividing peak area in “Exposure” with mean peak area in “Control”. Data are shown as mean ± standard deviation. Significantly different levels compared with related control are labeled with an asterisk (at *P* < 0.05) or two asterisks (at *P* < 0.01) based on a two-tailed student *t*-test.

In order to learn more about significantly changed metabolites after acute exposure, features with variable importance on projection (VIP) values larger than 1.5 and *P* values smaller than 0.05 in *t*-test were identified and shown in Table 3. These metabolites include eight amino acids (asparagine, leucine, lysine, phenylalanine, proline, serine, threonine and valine), glucose, inosine and seven phospholipids. Fold changes of these metabolites were shown in Fig. 4. For amino acids, glucose and inosine, levels at 1/5LC_50_-day2 are all nearly half of the levels at control-day2. In addition, the levels of these metabolites were elevated to varying extents at 1/10LC_50_-day1, 1/5LC_50_-day1 and 1/10LC_50_-day2 than the related control. Phospholipids with only one long fatty acid chain (lysophosphatidylcholine and lysophosphatidylethanolamine, LPC and LPE) exhibited significantly higher levels after exposure, while phospholipids with two long fatty acid chains (phosphatidylcholine, PC) showed significantly lower levels after exposure.

**Table 3:** Differentiating metabolites after acute exposure identified from GC-MS and LC-QTOF spectra.

Metabolite	Retention time (min)	Spectra	*m/z*	VIP	p value
asparagine	18.64	GC-MS	—	4.40	0.0008
leucine	12.07	GC-MS	—	3.58	0.0034
lysine	19.23	GC-MS	—	8.06	0.0090
phenylalanine	17.92	GC-MS	—	9.99	0.0118
proline	16.32	GC-MS	—	11.33	0.0180
serine	13.61	GC-MS	—	5.21	0.0184
threonine	14.07	GC-MS	—	4.30	0.0053
valine	11.02	GC-MS	—	6.28	0.0104
glucose	22.23	GC-MS	—	3.50	0.0054
inosine	29.76	GC-MS	—	6.26	0.0107
PE(P-16:0/0:0)	18.70	LC-QTOF positive mode	438.2969	2.23	0.0263
PC(P-14:0/0:0)	19.78	LC-QTOF positive mode	452.3124	3.53	0.0105
PC(O-15:0/O-0:0)	21.02	LC-QTOF positive mode	468.3429	2.42	0.0077
PC(O-16:1/1:0)	18.73	LC-QTOF positive mode	494.3588	1.84	0.0094
PC(O-16:0/1:0)	20.42	LC-QTOF positive mode	496.3025	1.71	0.0259
PC(O-15:0/24:6)	27.09	LC-QTOF positive mode	736.5259	1.79	0.0123
PC(O-15:0/24:5)	24.53	LC-QTOF positive mode	738.5407	1.74	0.0077

## Discussion

The acute exposure bioassay was designed as a filter paper test because it is time-saving, easily controllable, and allows direct contact of TPHP with earthworms. The chronic exposure experiment was conducted in soil to mimic practical scenarios in the environment. Although the concentrations used in the soil experiments exceeded those commonly reported environmentally relevant concentrations in the literature^20,21^, they allowed accumulation of TPHP metabolites that makes further identification easier using LC-QTOF and information dependent acquisition.

TPHP in soil enters the earthworm body likely through both dermal and ingestion routes. Bioaccumulation is the direct biological measure of bioavailability of chemicals in soil. The calculated BCFs of TPHP in soil-earthworm ecosystem are much smaller than the theoretical BCF of 113.3. This can be ascribed to TPHP degradation in soil, high hydrophobicity of TPHP which results in low concentration in pore water^32^, and metabolism of TPHP by the earthworms, which might be accelerated by gut microflora and ingested soil microflora. Quantitative data on the fate of TPHP in different matrices in the soil-earthworm ecosystem might be obtained in the future using isotope-labeled TPHP. Environmentally relevant bioaccumulation study of TPHP in the soil-earthworm ecosystem is also warranted.

Common enzymes in earthworms responsible for xenobiotic metabolism include cytochrome P450, carboxylesterases and glutathione-S-transferases (GST)^32^. For TPHP, oxidative hydroxylation is likely catalyzed by cytochrome P450, while hydrolysis may be catalyzed by phosphotriesterase. Previous work has indicated the presence of phosphotriesterase in *Eisenia andrei*^33^ and phosphodiesterase in *Lumbricus terrestris*^34^ being able to hydrolyze phosphor-triester and -diester pesticides, respectively. We did not observe degradation of diphenyl phosphate, possibly due to lack of phosphodiesterase in *P. excavatus*. Previously reported phase II conjugations of organic xenobiotics in earthworms include spermine^35^, glutamyl^36–38^ and glucoside^37,38^ conjugations. Our separate study (under peer review) revealed that phosphate was substantially used to conjugate hydroxylated tributyl phosphate (TBP) in both acute and chronic exposure scenarios^39^. This study reported glucoside and thiol conjugations of TPHP in *P. excavatus*. Glucoside conjugation is reasonable as it typically appears to be a more common route than glucuronic acid conjugation in invertebrates compared to vertebrates^40^. The phosphate-glucoside and C_14_H_19_O_10_P conjugates might be further metabolites of the glucoside conjugation. An alternative explanation might be that earthworm glucoside transferases are readily saturated, thus other similar enzymes may be used to metabolize excessive TPHP. The thiol conjugates include, but are not limited to, glutathione conjugates. Glutathione conjugation, catalyzed by GST, accounts for only a small portion of the thiol conjugates, while its hydrolysis products, cysteinylglycine conjugates and cysteine conjugates, account for larger portions. The activity of GST varies among different earthworm species^41^. Recent studies have shown that when *Aporrectodea caliginosa* and *E. andrei* were exposed to organophosphate pesticides, the activity of GST increased^42–44^. Our study revealed that *P. excavatus* may also possess GST activity to some extent. The presence of other thiol conjugates has not been reported previously in earthworms. These thiols may serve as complementary chemical pool to conjugate excessive xenobiotics when GST activity is limited. These findings indicate unique enzyme systems in *P. excavatus* for detoxicification of xenobiotics.

It should be noted that the identified phase II metabolites are putative structures and require further validation using nuclear magnetic resonance technique and commercial or synthesized metabolite standards. This is especially true for glucoside conjugates, because the conjugated molecules are large and complicated, and the fragments in MS/MS spectra are few and not informative. For example, the ion with *m/z* 583.0797 in negative mode has only one fragment with *m/z* 241.0118 in its MS/MS spectrum besides phosphate fragments. Another factor that makes unambiguous identification difficult is that the binding positions between different moieties could not be recognized via LC-QTOF. For example, the binding location of phosphate to the glucose structure is unknown. Nevertheless, these putative metabolites provide evidences of novel phase II metabolism of xenobiotics in *P. excavatus*.

Only acute exposure of TPHP for two days at 1/10 and 1/5 LC_50_ in filter-paper contact test resulted in significant perturbations of the endogenous metabolome in earthworms. The amino acids increased after one day exposure and decreased after two days exposure at 1/5 LC_50_. This concentration-dependent trend is similar to the perturbations of amino acids (glycine, valine, serine, phenylalanine, glutamine, lysine and tyrosine) in McKelvie *et al*.’s study^45^ after acute exposure of *E. fetida* to endosulfan, as well as the reported changes of amino acid levels (leucine, arginine, lysine and phenylalanine) after exposure of *E. fetida* to phenanthrene^46^. In stress conditions, such as exposure to high concentration of TPHP, increased utilization of glucose might occur due to high energy consumption^47^. Moreover, we have shown that glucose and its derivatives may be utilized to conjugate OH-TPHP. Thus, significant decrease of glucose after two days exposure at high concentration of TPHP is reasonable. Inosine, an intermediate in nucleotide synthesis, showed a similar trend as the amino acids and glucose, suggesting possible connection of their metabolic pathways. An earlier study showed that inosine can be generated by breakdown of 5′-adenosine monophosphate (5′-AMP) in the earthworm *L. terrestris*^48^. AMP can be used to synthesize adenosine diphosphate, a primary substrate for synthesis of adenosine triphosphate, the main product of glycolysis. Observed decrease of inosine in this study might be the result of higher glycolytic activity for enzyme production in response to excessive TPHP body-burden. Similar associations of amino acids, glucose and inosine have also been observed in earthworms after exposure to C_60_ nanoparticles^49^. Phospholipids are major components of cell membrane, and the lipophilic nature of TPHP makes it easily retained in the cell membrane, causing membranes to swell and increasing lipid membrane fluidity^50,51^. The perturbations of phospholipid metabolism might result from maintenance of cell membrane integrity. Moreover, significant decrease of PC is accompanied by significant increase of lysophosphatidylcholine bearing the same fatty acid chain, possibly suggesting increased hydrolysis of phosphatidylcholine arising from enhanced phospholipase A2 activity^52,53^.

The metabolomics results in soil experiments indicated no significant differences of endogenous metabolome between the control and exposure groups. It can be inferred that TPHP may not be toxic to earthworms under environmentally relevant concentrations, at least at the metabolic level.

In summary, the bioaccumulation, biotransformation and perturbation of endogenous metabolites of earthworms after exposure to TPHP using a filter-paper contact test and soil microcosm experiment revealed that the bioaccumulation potential of TPHP in earthworm-soil ecosystem is low at 10 mg/kg and 50 mg/kg of TPHP. Various phase I and phase II biotransformation products were putatively identified in the earthworms. Phase I metabolites include DPHP, OH-TPHP and di-OH-TPHP, while phase II metabolites can be categorized to thiol conjugates and glucoside conjugates. These metabolites reveal novel phase II metabolism of xenobiotics in *P. excavatus*. Only acute TPHP exposure caused significant perturbations of the endogenous metabolome in earthworms, featuring fluctuations in amino acids, glucose, inosine and phospholipids. Alterations of metabolites might be useful for understanding toxic mode of action of TPHP following acute exposure.

## Materials and Methods

### Earthworm culture

The tropical composting earthworm^22^ species *Perionyx excavatus* was employed in this study. The worms were purchased from a local worm farm in Singapore. Upon collection, worms were placed in soil and fed with sheep manure for one month at 20 ± 1 °C in laboratory for adaptation. Mature worms with clear clitellum were used in all experiments. These worms had weights ranging from 0.65 g to 1.12 g, and lengths ranging from 13 cm to 17 cm.

### LC_50_ test

The LC_50_ test was first conducted based on the OECD guideline on acute toxicity test using earthworms^54^. Briefly, 1 ml solutions of TPHP (Sigma-Aldrich, CAS 115-86-6) in acetone were pipetted onto a filter paper disk of 12.5 cm^2^ placed in amber glass jars. A geometric series of concentrations of 0.000128, 0.00128, 0.0128, 0.128, 1.28 mg/cm^2^ TPHP were prepared with 10 replicates. The filter papers were placed in a fume hood for 4 h to allow acetone evaporation before earthworm dermal exposure under dark conditions. Based on the recorded mortalities after 48 h exposure under these concentrations, another two medium concentrations of 0.0043 and 0.0085 mg/cm^2^ were further tested using the same protocol.

### Acute exposure

The acute exposure assay was conducted on filter paper disks similar to the LC_50_ test. LC_50_ was first calculated as 4.25 μg/cm^2^ (Supplementary Fig. S1). TPHP concentrations in the acute exposure experiment were set as 1/10 and 1/5 of the LC_50_, i.e. 0.425 and 0.85 μg/cm^2^, respectively. The control consisted of solvent applied to filter paper disks. Worms were collected after exposure of 24 h and 48 h. Eight replicates were prepared and in each replicate one worm was used.

### Chronic exposure

Chronic exposure was conducted in soil microcosm using a silty clay loam soil collected from the campus of National University of Singapore. The soil pH was 7.8 and organic matter content was 7.7%. After stones and plant residues were removed from the soil, the soil was air dried, ground and sieved through a 1-mm sieve before being used in assays.

Acetone (0.2 ml) containing TPHP was dispensed onto 20 g of soil, which was then placed in fume hood for 30 min to allow solvent evaporation. Concentrations of 0, 10 and 50 mg TPHP per kg were prepared by thorough mixing of the spiked soil with 80 g clean soil. Water was added to the prepared soils to reach 25% (weight percentage) before adding worms. All glass beakers were placed in the dark at 18 °C. There were 8 replicates in each concentration, and one worm was used in each replicate. Worms were fed with 5 g sheep manure containing 0, 10 or 50 mg/kg TPHP every 7 days. Soil spiked with TPHP without earthworms and with addition of sheep manure was prepared with three replicates in the same protocol. Worms were collected after 28 days exposure, rinsed and placed onto a wet filter paper in a beaker for 24 h in the dark to empty the gut contents. Three replicates of soil with and without earthworms were sampled at day 0, 1, 2, 3, 5, 8, 13, 21 and 28 after TPHP spiking and kept at −80 °C until extraction.

### Simultaneous extraction of TPHP metabolites and endogenous metabolites of earthworm

Collected worms were all flash-frozen and ground in liquid nitrogen using mortar and pestle, then kept at −80 °C until extraction. To simultaneously extract TPHP metabolites and endogenous metabolites of earthworm, 600 μl ice cold methanol (LC-MS grade)/ultrapure water (volume ratio 4:1) containing internal standard N-9-Fmoc-L-glycine (20 mg/l, Sigma Aldrich, CAS 29022-11-5, >98%) was added to 50 mg worm tissue. The mixture was homogenized with three zirconium oxide beads (2.8 mm diameter) at medium speed for 60 s and two times using a Minilys homogenizer (Bertin Technologies, France). Then, the mixture was vortexed for 5 min, placed on dry ice for 15 min, and centrifuged at 14000 rpm for 10 min at 4 °C. The supernatant was transferred to a sample vial and analysed by LC-QTOF. An aliquot of 50 μl was dried in a vacuum concentrator (Labconco, MO, USA) at 4 °C for further derivatization and analysis via GC-MS.

### GC-MS derivatization and analysis

The dried extract was mixed with 50 μl methoxyamine hydrochloride (15 mg/ml in pyridine, Sigma Aldrich) and incubated at 30 °C for 90 min. After adding 100 μl N,O-Bis(trimethylsilyl)trifluoroacetamide containing 1% trimethylsilyl chloride (Sigma Aldrich), the mixture was further incubated at 60 °C for 1 h. The derivatized sample was analysed using a GC (Agilent 7890A), coupled with a triple quadrupole mass analyser (Agilent 7000B). The ion source temperature, transfer line temperature and injector temperature were set at 230 °C, 290 °C and 250 °C, respectively. The oven temperature ramp was set as follows: initially at 60 °C for 2 min, increased to 300 °C at a rate of 7.5 °C/min and held for 6 min. The carrier gas was helium at a flow rate of 1.0 ml/min. Derivatized samples of 1 μl were injected via splitless mode, and analyzed under full scan mode. Metabolites were separated on a DB-5MS capillary column (30 m X 0.25 mm i.d., 0.25 μm film thickness) consisting of a stationary phase of 5% phenyl 95% methylpolysiloxane, and detected in electron impact ionization mode at 70 eV. Acquired mass range was 50 – 650 Da. Samples were randomized and analysed within 24 h after derivatization.

### LC-QTOF analysis

The extracted supernatants were analysed using a Dionex Ultimate 3000 HPLC system (Thermo Fisher Scientific, USA) coupled to a TripleTOF 5600 system (AB Sciex, USA) with electrospray ionization (ESI) source under both positive and negative mode. In positive mode, mobile phase A and B were 0.1% formic acid in water and acetonitrile, respectively, while in negative mode, mobile phase A was changed to 10 mM ammonium formate in water. The injection volume was 3 μl. Chromatographic separation was achieved with a Zorbax Eclipse Plus C_18_ column (2.1 × 100 mm, 3.5 μm) at flow rate of 0.3 ml/min using the following gradient: 5% B for 2 min, ramped to 95% B at 22 min, held for 2 min, then dropped to 5% B over 0.1 min and held for 6 min. TOF scan was performed simultaneously with product ion scan using information dependent acquisition (IDA). In each acquisition cycle, ten of the most abundant ions were chosen for MS/MS experiments at collision energy of 35 eV and spread of 15 eV. The accumulation time for TOF scan and product ion scan was set as 250 ms and 100 ms, respectively. The ESI source parameters were as follows: nebulizer gas = 50 psi, heater gas = 50 psi, curtain gas = 25 psi, and heater temperature = 550 °C. The ion spray voltage and declustering potential were 5400 and 80, respectively, in positive mode and −4500 and −100 in negative mode. Acquired mass range was set as 50–1000 Da for both TOF and product ion scan. The samples were randomized and analysed at 4 °C. Each batch was run within 12 h to ensure sample stability based on a preliminary test.

### Determination of TPHP in earthworm and soil

TPHP in soil and earthworm was extracted using acetonitrile with recovery of 87–95%. Soils and earthworms were dried in a freeze dryer (Labconco, MO, USA). Dry soil or earthworm tissue (0.2 g) was mixed with 1 ml acetonitrile, sonicated for 30 min and centrifuged. This process was repeated and resulting supernatants were pooled. The extracts were diluted to a pre-determined calibration range and analysed using a Dionex Ultimate 3000 HPLC coupled with a QTrap 5500 system (AB Sciex, USA) with ESI source under positive mode. Mobile phases used were 0.1% formic acid in water (A) and 0.1% formic acid in acetonitrile (B). Gradient was set at 70% B at 0–2 min, increased to 100% B in 3 min, then dropped to 70% B in 0.1 min and kept for 6 min. Two *m/z* transitions were used for quantitation and confirmation: 327 → 152 (CE 52 eV, CXP 18 eV) and 327 → 215 (CE 33 eV and CXP 12 eV) at DP 150 eV and EP 8 eV.

### Data processing and statistical analysis

The GC-MS and LC-QTOF data files were converted to mzXML format and uploaded to XCMS online (https://xcmsonline.scripps.edu) to extract features. Intensities of internal standard were used to normalize feature intensities in LC-QTOF data. Features from blanks with average intensity larger than half of the average intensity from samples were removed. Multivariate and univariate analysis were performed in the online MetaboAnalyst platform^55^. Pareto scaling was performed before PCA and PLS-DA. Features with VIP values in PLS-DA larger than 1.5 were selected and further subjected to student *t*-test in the same platform. Only significantly different features (*P* < 0.05) in the *t*-test were identified as corresponding metabolites together with associated isotopes, adducts and fragments.

### Metabolites identification

We used several approaches to screen potential TPHP metabolites in LC-QTOF spectra. First, literature reported phase I TPHP metabolites were screened. From their MS/MS spectra, we found that 215.0245 is a typical and abundant fragment in positive mode. Second, we screened 215.0245 in product ion spectra obtained by IDA. Ions with similar MS/MS spectra with TPHP were identified, including thiol conjugates. We further screened 357.0335 using the same strategy in search of more thiol conjugates. Glucoside conjugates were identified in the same manner in negative mode. Third, *t*-tests between features extracted from exposure and control spectra were performed. Features with low *P* values (<0.001) were selected. Last, we also employed selected ion extraction based on suspected structures to find low intensity metabolites for which the data-dependent MS/MS were not triggered. In these approaches, compounds present in control without TPHP spiking were removed. Suspected ions were putatively identified using the following four criteria: (1) the accuracy of *m/z* values of precursor ions does not exceed 5 ppm; (2) the isotope ratios are similar as theoretical values; (3) fragments in MS/MS spectra can be explained; (4) retention time is reasonable based on polarity of a proposed structure.

The earthworm endogenous metabolites in the GC-MS spectra were identified using National Institute of Standards and Technology library. Only metabolites with matching scores of >70% were considered as reliable. Endogenous metabolites in LC-QTOF dataset were identified using accurate *m/z* of the precursor ion combined with comparison of the corresponding MS/MS spectrum in the METLIN MS/MS spectral database.

## Electronic supplementary material

Supplementary Information

## Data Availability

The datasets generated during and analysed during the current study are available from the corresponding author on reasonable request.

## References

[1] Wei GL (2015). Organophosphorus flame retardants and plasticizers: Sources, occurrence, toxicity and human exposure. Environ. Pollut..

[2] Van der Veen I, de Boer J (2012). Phosphorus flame retardants: Properties, production, environmental occurrence, toxicity and analysis. Chemosphere.

[3] McGee SP, Konstantinov A, Stapleton HM, Volz DC (2013). Aryl phosphate esters within a major pentaBDE replacement product induce cardiotoxicity in developing zebrafish embryos: Potential role of the aryl hydrocarbon receptor. Toxicol. Sci..

[4] Stapleton HM (2009). Detection of Organophosphate Flame Retardants in Furniture Foam and US House Dust. Environm. Sci. Technol..

[5] Dodson RE (2012). After the PBDE Phase-Out: A Broad Suite of Flame Retardants in Repeat House Dust Samples from California. Environ. Sci. Technol..

[6] Marklund A, Andersson B, Haglund P (2005). Traffic as a source of organophosphorus flame retardants and plasticizers in snow. Environ. Sci. Technol..

[7] Green, N. *et al*. Screening of Selected Metals and New Organic Contaminants 2007. *NIVA Report* 5569–2008 (2008).

[8] Su GY, Crump D, Letcher RJ, Kennedy SW (2014). Rapid *in Vitro* Metabolism of the Flame Retardant Triphenyl Phosphate and Effects on Cytotoxicity and mRNA Expression in Chicken Embryonic Hepatocytes. Environ. Sci. Technol..

[9] Su GY, Letcher RJ, Crump D, Gooden DM, Stapleton HM (2015). *In Vitro* Metabolism of the Flame Retardant Triphenyl Phosphate in Chicken Embryonic Hepatocytes and the Importance of the Hydroxylation Pathway. Environ. Sci. Technol. Let..

[10] Van den Eede N, Maho W, Erratico C, Neels H, Covaci A (2013). First insights in the metabolism of phosphate flame retardants and plasticizers using human liver fractions. Toxicol. Let..

[11] Van den Eede N, Ballesteros-Gomez A, Neels H, Covaci A (2016). Does Biotransformation of Aryl Phosphate Flame Retardants in Blood Cast a New Perspective on Their Debated Biomarkers?. Environ. Sci. Technol..

[12] Wang GW (2016). Tissue-Specific Accumulation, Depuration, and Transformation of Triphenyl Phosphate (TPHP) in Adult Zebrafish (Danio rerio). Environ. Sci. Technol..

[13] Zhang Q (2014). Potential Estrogenic Effects of Phosphorus-Containing Flame Retardants. Environ. Sci. Technol..

[14] Liu X, Ji K, Jo A, Moon HB, Choi K (2013). Effects of TDCPP or TPP on gene transcriptions and hormones of HPG axis, and their consequences on reproduction in adult zebrafish (Danio rerio). Aquat. Toxicol..

[15] Liu X, Ji K, Choi K (2012). Endocrine disruption potentials of organophosphate flame retardants and related mechanisms in H295R and MVLN cell lines and in zebrafish. Aquat. Toxicol..

[16] Liu XS (2016). Long-term exposure to triphenyl phosphate alters hormone balance and HPG, HPI, and HPT gene experission in zebrafish (*Danio rerio*). Environ. Toxicol. Chem..

[17] Kim S (2015). Thyroid disruption by triphenyl phosphate, an organophosphate flame retardant, in zebrafish (Danio rerio) embryos/larvae, and in GH3 and FRTL-5 cell lines. Aquat. Toxicol..

[18] Sun LW (2016). Developmental neurotoxicity of organophosphaate flame retardants in early life stages of Japanese medaka (*Oryzias latipes*). Environ.Toxicol. Chem..

[19] Morris PJ (2014). Organophosphorus Flame Retardants Inhibit Specific Liver Carboxylesterases and Cause Serum Hypertriglyceridemia. ACS Chem. Biol..

[20] Matsukami H (2015). Flame retardant emission from e-waste recycling operation in northern Vietnam: Environmental occurrence of emerging organophosphorus esters used as alternatives for PBDEs. Sci. Total Environ..

[21] Wan WN, Zhang SZ, Huang HL, Wu T (2016). Occurrence and distribution of organophosphorus esters in soils and wheat plants in a plastic waste treatment area in China. Environ. Pollut..

[22] Edwards, C. A. *Earthworm ecology*. Vol. 2nd (CRC Press, 2004).

[23] Bundy JG, Davey MP, Viant MR (2009). Environmental metabolomics: a critical review and future perspectives. Metabolomics.

[24] Aslund MW, Simpson MJ, Simpson AJ, Zeeb BA, Rutter A (2012). Earthworm metabolomic responses after exposure to aged PCB contaminated soils. Ecotoxicology.

[25] Yuk J, Simpson MJ, Simpson AJ (2012). Coelomic fluid: a complimentary biological medium to assess sub-lethal endosulfan exposure using ^1^H NMR-based earthworm metabolomics. Ecotoxicology.

[26] Aslund MLW (2012). Earthworm Sublethal Responses to Titanium Dioxide Nanomaterial in Soil Detected by H-1 NMR Metabolomics. Environ. Sci. Technol..

[27] Du, Z. K. *et al*. TPhP exposure disturbs carbohydrate metabolism, lipid metabolism, and the DNA damage repair system in zebrafish liver. *Sci. Reports* **6** (2016). 10.1038/srep21827 PMC4761896 26898711

[28] Su GY, Letcher RJ, Yu HX (2016). Organophosphate Flame Retardants and Plasticizers in Aqueous Solution: pH-Dependent Hydrolysis, Kinetics, and Pathways. Environ. Sci. Technol..

[29] Anderson C, Wischer D, Schmieder A, Spiteller M (1993). Fate of triphenyl phosphate in soil. Chemosphere.

[30] Lenz EM, Lindon JC, Nicholson JK, Weeks JM, Osborn D (2002). F-19-NMR and directly coupled F-19/H-1-HPLC-NMR spectroscopic investigations of the metabolism of the model ecotoxin 3-trifluoromethylaniline in the earthworm species *Eisenia veneta*. Xenobiotica.

[31] Gromski PS (2015). A tutorial review: Metabolomics and partial least squares-discriminant analysis - a marriage of convenience or a shotgun wedding. Analytica Chimica Acta.

[32] Katagi T, Ose K (2015). Toxicity, bioaccumulation and metabolism of pesticides in the earthworm. J. Pestic. Sci..

[33] Sik Lee M (2001). Partial characterization of phosphotriesterase activity from the earthworm, Eisenia andrei. Int. Biodeter. Biodegr..

[34] Park SC, Smith TJ, Bisesi MS (1993). Bioactivation of BIS[p-nitrophenyl]phosphate by phosphoesterases of the earthworm: Lumbricus terrestris. Drug Chem. Toxicol..

[35] Curl EA, Edwards PJ, Elliott C, LeAhey JP (1987). The conjugation and accumulation of metabolites of cypermethrin by earthworms. Pestic. Sci..

[36] Sloley BD (1994). γ-Glutamyl conjugation of 5-hydroxytryptamine (serotonin) in the earthworm (*Lumbricus terrestris*). Neurochem. Res..

[37] Duckett CJ (2007). Metabolism of 2-fluoro-4-iodoaniline in earthworm *Eisenia veneta* using 19F-NMR spectroscopy, HPLC-MS, and HPLC-ICPMS (127I). Xenobiotica.

[38] Bundy JG (2002). Metabolism of 4-fluoroaniline and 4-fluorobiphenyl in the earthworm *Eisenia veneta* characterized by high-resolution NMR spectroscopy with directly coupled HPLC-NMR and HPLC-MS. Xenobiotica.

[39] Wang L, Huang XL, Laserna AKC, Li SFY (2018). Metabolism of tri-*n*-butyl phosphate in earthworm *Perionyx excavatus*. Environmental Pollution.

[40] Livingstone DR (1998). The fate of organic xenobiotics in aquatic ecosystems: quantitative and qualitative differences in biotransformation by invertebrates and fish. Comp. Biochem. Phys. A.

[41] Stenersen J, Guthenberg C, Mannervik B (1979). Glutathione S-transferases in earthworms (Lumbricidae). Biochem. J..

[42] Stepic S, Hackenberger BK, Velki M, Loncaric Z, Hackenberger DK (2013). Effects of individual and binary-combined commercial insecticides endosulfan, temephos, malathion and pirimiphos-methyl on biomarker responses in earthworm *Eisenia andrei*. Environ. Toxicol. Phar..

[43] Velki M, Hackenberger BK (2013). Inhibition and recovery of molecular biomarkers of earthworm *Eisenia andrei* after exposure to organophosphate dimethoate. Soil Biol. Biochem..

[44] Sanchez-Hernandez JC, Narvaez C, Sabat P, Mocillo SM (2014). Integrated biomarker analysis of chlorpyrifos metabolism and toxicity in the earthworm *Aporrectodea caliginosa*. Sci. Total Environ..

[45] McKelvie JR, Yuk J, Xu YP, Simpson AJ, Simpson MJ (2009). ^1^H NMR and GC/MS metabolomics of earthworm responses to sub-lethal DDT and endosulfan exposure. Metabolomics.

[46] Lankadurai BP, Wolfe DM, Aslund MLW, Simpson AJ, Simpson MJ (2013). ^1^H NMR-based metabolomic analysis of polar and non-polar earthworm metabolites after sub-lethal exposure to phenanthrene. Metabolomics.

[47] Ch, R., Singh, A. K., Pandey, P., Saxena, P. N. & Mudiam, M. K. R. Identifying the metabolic perturbations in earthworm induced by cypermethrin using gas chromatography-mass spectrometry based metabolomics. *Sci. Reports* **5** (2015). 10.1038/srep15674 PMC4626786 26514086

[48] Macdonnell PC, Tillinghast EK (1973). Metabolic sources of ammonia in earthworm. Lumbricus-terrestrial. J. Exp. Zool..

[49] Lankadurai BP, Nagato EG, Simpson AJ, Simpson MJ (2015). Analysis of *Eisenia fetida* earthworm responses to sub-lethal C-60 nanoparticle exposure using ^1^H-NWIR based metabolomics. Ecotoxicol. Environ. Safety.

[50] Mackay D (2009). The physicochemical basis of QSARs for baseline toxicity. SAR QSAR Environ. Res..

[51] Ankley GT (2010). Adverse outcome pathways: A conceptural framework to support ecotoxicology research and risk assessment. Environ. Toxicol. Chem..

[52] García-Sevillano MA (2014). Use of metallomics and metabolomics to assess metal pollution in Doñana National Park (SW Spain). Environ. Sci. Technol..

[53] Schomburg, D. & Michal, G. *Biochemical pathways: an atlas of biochemistry and molecular biology*. Vol. 2nd (John Wiley & Sons, 2012).

[54] OECD. Test No. 207: Earthworm, Acute Toxicity Tests. (OECD Publishing).

[55] Xia JG, Sinelnikov IV, Han B, Wishart DS (2015). MetaboAnalyst 3.0-making metabolomics more meaningful. Nucleic Acids Res..

