# Supplementary material for: Untargeted metabolomics reveals transformation pathways and metabolic response of the earthworm Perionyx excavatus after exposure to triphenyl phosphate
Source: Sci Rep. 2018 Nov 6;8:16440. doi: 10.1038/s41598-018-34814-9 (PMC6219545; doi:10.1038/s41598-018-34814-9)
Supplement: Supplementary file 1 — Supplementary Information [file 41598_2018_34814_MOESM1_ESM.pdf]

Supplementary Information

Untargeted metabolomics reveals transformation pathways and metabolic responses of the earthworm *Perionyx excavatus* after exposure to triphenyl phosphate

Lei Wang[1], Xulei Huang[1], Anna Karen Carrasco Laserna[1] and Sam Fong Yau Li[1,2,*]

[1]Department of Chemistry, National University of Singapore, 3 Science Drive 3, Singapore.

[2]NUS Environmental Research Institute (NERI), #02-01, T-Lab Building (TL), 5A Engineering Drive 1, Singapore 117411, Singapore.

[*]Correspondence and requests for materials should be addressed to S.F.Y. Li. (email: chmlifys@nus.edu.sg)

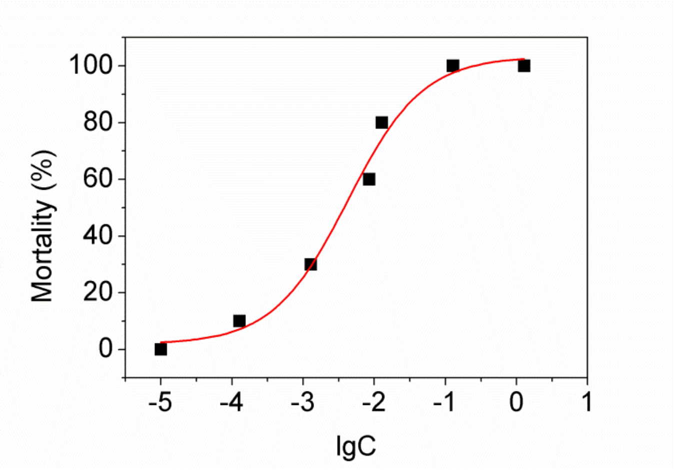

Supplementary Figure S1 Earthworm mortalities after dermal exposure for 48 h to filter papers with a series of TPHP concentrations. C: concentrations expressed as μg TPHP per cm^2 filter paper.

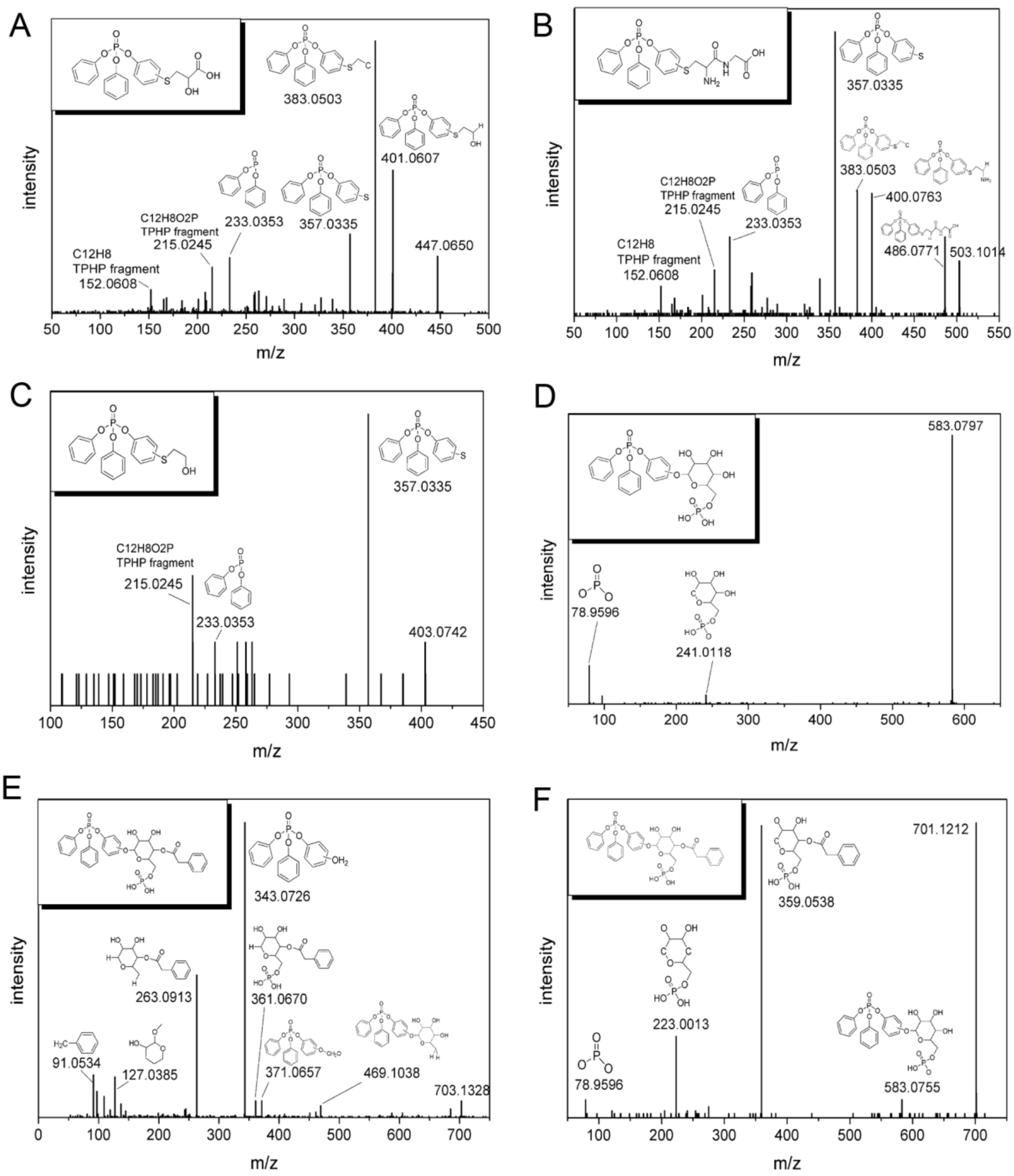

Supplementary Figure S2 MS/MS spectrum of (A) mercaptolactic acid-TPHP (*m/z* 447.0658 in ESI+), (B) cysteinylglycine-TPHP (*m/z* 503.1014 in ESI+), (C) mercaptoethanol-TPHP (*m/z* 403.0742 in ESI+), (D) phosphate-glucoside-TPHP (*m/z* 583.0797 in ESI-), and $C_{14}H_{19}O_{10}P$-TPHP [(E) *m/z* 703.1316 in ESI+ and (F) *m/z* 701.1212 in ESI-] with annotation of the putative fragments.

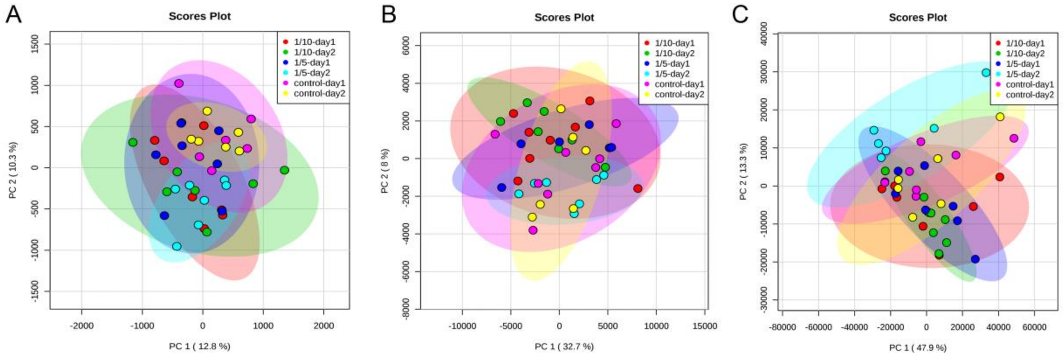

Supplementary Figure S3 Principal component analysis of extracted features from LC-QTOF spectra in (A) positive mode and (B) negative mode, and from (C) GC-MS spectra after acute exposure. The labels 1/10 and 1/5 refer to 1/10 LC50 and 1/5 LC50 exposure groups, respectively.

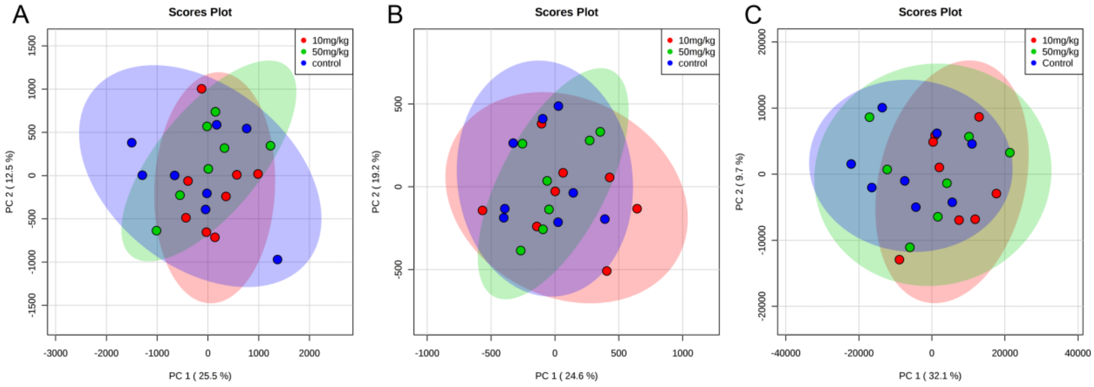

Supplementary Figure S4 Principal component analysis of extracted features from LC-QTOF spectra in (A) positive mode and (B) negative mode, and from (C) GC-MS spectra after chronic exposure.

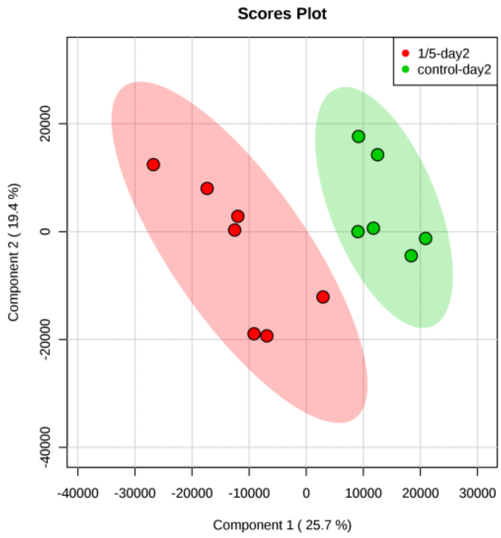

Supplementary Figure S5 Score plot of partial least squares discriminant analysis of GC-MS data (Control-day 2 versus 1/5 LC_{50}-day2) after probabilistic quotient normalization.
